# The gut microbiome in differential diagnosis of diabetic kidney disease and membranous nephropathy

**DOI:** 10.1080/0886022X.2020.1837869

**Published:** 2020-10-30

**Authors:** Wei Yu, Jin Shang, Ruixue Guo, Fanliang Zhang, Weifeng Zhang, Yiding Zhang, Feng Wu, Hongyan Ren, Chao Liu, Jing Xiao, Zhanzheng Zhao

**Affiliations:** a Department of Nephrology, The First Affiliated Hospital of Zhengzhou University, Zhengzhou, P.R. China; b Shanghai Mobio Biomedical Technology Co, Ltd, Shanghai, P.R. China

**Keywords:** Gut microbiome, 16S rRNA, diabetic kidney disease, membranous nephropathy

## Abstract

**Background:**

Diabetic kidney disease (DKD) and membranous nephropathy (MN) are the two major causes of end-stage renal disease (ESRD). Increasing evidence has shown that intestinal dysbiosis is associated with many diseases. The aim of this study was to explore the composition of the gut microbiome in DKD and MN patients.

**Methods:**

16S rRNA gene sequencing was performed on 271 fecal samples (DKD = 129 and MN = 142), and taxonomic annotation of microbial composition and function was completed.

**Results:**

We observed distinct microbial communities between the two groups, with MN samples exhibiting more severe dysbiosis than DKD samples. Relative increases in genera producing short-chain fatty acids (SCFAs) in DKD and a higher proportion of potential pathogens in MN were the main contributors to the microbiome alterations in the two groups. Five-fold cross-validation was performed on a random forest model, and four operational taxonomic unit (OTU)-based microbial markers were selected to distinguish DKD from MN. The results showed 92.42% accuracy in the training set and 94.52% accuracy in the testing set, indicating high potential for these microbiome-based markers in separating MN from DKD. Overexpression of several amino acid metabolic pathways, carbohydrate metabolism and lipid metabolism was found in DKD, while interconversion of pentose/glucoronate and membrane transport in relation to ABC transporters and the phosphotransferase system were increased in MN.

**Conclusion:**

The composition of the gut microbiome appears to differ considerably between patients with DKD and those with MN. Thus, microbiome-based markers could be used as an alternative tool to distinguish DKD and MN.

## Introduction

As the common cause of primary or secondary glomerular disease worldwide, membranous nephropathy (MN) and diabetic kidney disease (DKD) have similar clinical manifestations characterized by nephrotic syndrome [1,2]. The incidence of DKD has become an epidemic in the past decade, mainly driven by the increasing global prevalence of diabetes mellitus (DM) [3]. Increasing evidence has revealed that DKD is the leading cause (approximately 50%) of the progression of chronic kidney disease (CKD) to end-stage renal disease (ESRD) worldwide [4], followed by MN at 30% [5]. Although clinical indicators relying on the excretion of urinary albumin and the course of DM (including those described in our previous study) have played a key role in DKD identification and prognostic prediction, the deficiency in early diagnosis and lack of specificity in differential diagnosis of DKD still urgently need to be remedied [6,7]. Renal biopsy cannot be performed frequently in DKD cases due to its invasiveness, which results in damage to patients [8]. Likewise, many cases of MN (approximately 20%) cannot be identified, as they are associated with negative responses to phospholipase A2 receptor (PLA2R)- and thrombospondin type 1 domain-containing protein 7 A (THSD7A)-targeted antigens [9–11]. Thus, this study attempts to explore a new approach to DKD/MN identification that offers broader coverage.

Recent studies have reported gut-microbiome-associated advances in the diagnosis, pathogenesis and treatment of DM [12,13] and DKD [14–17]. For instance, a gut-microbiota-targeted fiber diet could reduce insulin resistance and improve hyperglycemic status in people with diabetes [18,19]. In a mouse model of DKD, Li and his colleagues demonstrated that the genus *Blautia* had a protective effect on renal function progression from microalbuminuria to macroalbuminuria [20]. Inhibition of the synthesis of phenol sulfate, produced by fermentation of tyrosine in the gut, could lower the level of urinary protein in mice with DKD [21]. Notably, Tao’s study proposed the idea of gut-microbiome-based biomarkers in differential diagnosis of DKD and DM with high accuracy [22]. Although the role of the gut microbiome in a DKD mouse model has been studied, its function still needs to be verified in human subjects. Furthermore, the role of the intestinal flora in MN remains unclear.

Currently, kidney biopsy is the gold standard for distinguishing these two diseases. However, renal biopsy cannot be performed on a substantial number of patients due to contraindications, such as blood coagulation disorders. In addition, a subset of patients are unwilling to undergo kidney biopsy. In this circumstance, there is no effective way to provide diagnostic evidence to differentiate DKD patients from MN patients.

Our research group previously performed a preliminary exploration of microbial differences among 22 patients with diabetic nephropathy (DN) and 22 patients with MN (unpublished data). However, the diagnostic potential for microbial markers to identify DN from MN could not be confirmed due to a limited sample size. Thus, in the current study, we characterized compositional and functional changes in the gut microbiomes of 129 DKD patients and 142 MN patients and tried to establish an accurate discrimination index for DKD and MN.

## Material and methods

### Study cohort

One hundred twenty-nine patients with clinically diagnosed DKD and 142 patients with pathologically diagnosed MN were recruited from the First Affiliated Hospital of Zhengzhou University from January to December 2019. In accordance with the Declaration of Helsinki, our study was approved by the Institutional Review Board of the First Affiliated Hospital of Zhengzhou University (2019-KY-361). All participants signed an informed consent before sample collection.

The diagnostic criteria of DKD were based on at least two clinical indexes: more than 5 years history of diabetes and a ratio of urinary protein to creatinine ≥30 mg/g [23,24]. These patients also had diabetic retinopathy. All the MN cases were confirmed by renal biopsy [25]. Both groups excluded patients with gastrointestinal, tumor-associated diseases, viral hepatitis, cardiovascular or central nervous system diseases and other autoimmune diseases such as antineutrophil cytoplasmic antibody (ANCA)-associated vasculitis. Patients treated with antibiotics, prebiotics or nephrotoxic drugs within 6 months or with a history of surgery were also excluded. In addition, DKD patients with positive expression of PLA2R antibody were excluded. The biopsy results were assessed by two pathologists. All the participants enrolled were randomly divided into a discovery cohort and a validation cohort, as described below.

### Fecal sample collection and DNA extraction

At least 1 g of fresh, solid intestinal excretion was provided by individuals within 2 h, deposited into sterile tubes and frozen at −80 °C for further analysis. Fecal DNA was extracted as previously described [26]. Briefly, 790 µL sterile lysis buffer (containing 5% N-lauroyl sarcosine-0.1 mmol/l phosphate buffer [pH 8.0], 500 µL; 4 mmol/L guanidine thiocyanate, 250 µL; 10% N-lauroyl sarcosine, 40 µL) was added to fecal samples with the same mass; the mixture was incubated at 70 °C for 1 h after vigorous vortexing; the samples were beaten for 10 min with 1 g of glass beads to fully lyse the membranes of the cells and nuclei; final bacterial DNA extraction was completed using the E.Z.N.A. Stool DNA Kit (Omega Bio-tek, Inc., GA) following the manuscript’s protocols. The total obtained DNA was quality-controlled for PCR amplification.

### PCR amplification and 16S rRNA gene sequencing analysis

The V3-V4 region of the extracted DNA was amplified for DNA library construction and MiSeq sequencing. The general primers were as follows: F1, 5′- CCTACGGGNGGCWGCAG −3′, and R2, 5′-GACTACHVGGGTATCTAATCC-3′. PCR was performed with the following program: 3 min of predenaturation at 95 °C; followed by 21 cycles of 0.5 min denaturation at 94 °C, 0.5 min annealing at 58 °C, and 0.5 min elongation at 72 °C; and a final 5 min extension at 72 °C. The purified PCR products were mixed for MiSeq sequencing by Shanghai Mobio Biomedical Technology Co., Ltd. Sequences with zero mismatches were extracted for further quality filtering. Sequences with overlap lower than 50 bp, error rate of overlap higher than 0.1 and merged length lower than 400 bp were discarded using USEARCH 8.0 [27].

### OTU clustering and annotation

Quantity-controlled sequences obtained from the DKD and MN samples were separately sorted by abundance to identify representative sequences using the UPARSE pipeline [28]. The gene sequences were clustered into OTUs at 97% identity, and the phylogenetic affiliation of each OTU was annotated with RDP Classifier [29] against the Silva (SSU123)16S rRNA database [30] with a 70% confidence threshold.

### Bioinformatic and statistical analysis

We used rarefaction curves and species accumulation curves to ensure that the sample size or sequencing depth had reached saturation in our study. Bacterial α-diversity in the discovery cohort was shown by the Ace/Chao-based richness index and Shannon/Simpson-based diversity index. Principal coordinate analysis (PCoA) and nonmetric multidimensional scaling (NMDS) plots were generated to visualize the weighted UniFrac distances using Quantitative Insights into Microbial Ecology (QIIME) [31]. Corresponding statistical significance was determined by analysis of similarities (ANOSIM) and Adonis. Compositional differences between the two groups from the phylum to genus level were tested with a nonparametric Mann-Whitney U test. Variation at the taxonomic level was determined by Linear Discriminant Analysis (LDA) Effect Size (LEfSe) (LDA score >2.0).

The Wilcoxon rank-sum test was used to identify markedly different OTUs between DKD and MN, based on which 397 OTUs (*p* < 0.05) were incorporated into a random forest model to evaluate importance. Five trials with fivefold cross-validation were used to identify optimal combinations of microbial markers using 45 key OTU-abundance profiles from the discovery cohort (OTU importance >0.001). As a result, 96 DKD and 98 MN samples were randomly divided into the training set, and the remaining participants were incorporated into the testing set. Based on this diagnostic model, the possibility of disease (POD) of each sample was calculated to construct a receiver operating characteristic curve (ROC) for the two sets. Identification markers with an area under the ROC curve (AUC) greater than 0.7 were considered successful.

The potential bacterial metabolism in the two groups was predicted with Phylogenetic Investigation of Communities by Reconstruction of Unobserved States (PICRUSt) [32]. Significant differences in Kyoto Encyclopedia of Genes and Genomes (KEGG) pathways between DKD and MN were evaluated with LEfSe (LDA > 2.0 and *P* value < 0.05). All of the above analyses were completed in R (<seurld>http://www.R-project.org/</seurld>).

## Results

### Study cohort

**Figure 1.**
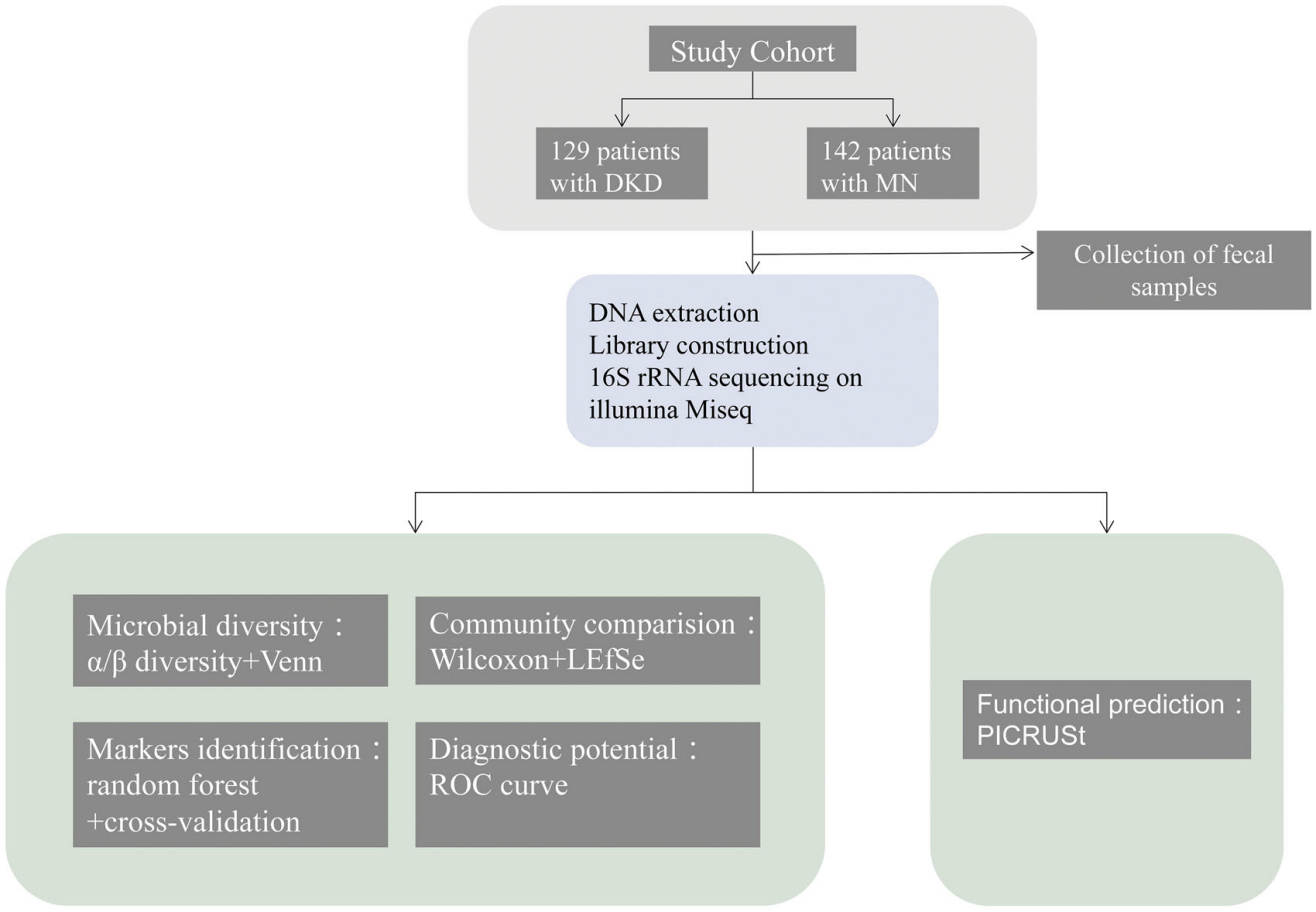
Study design. DKD, diabetic kidney disease; MN, membranous nephropathy; OTUs, operational taxonomic units.

A total of 129 patients with clinically diagnosed DKD and 142 patients with biopsy-proven MN were enrolled and incorporated into the discovery cohort (Figure 1). 16S rRNA gene sequencing data were obtained to characterize compositional and functional changes in the gut microbiome in both groups. Microbial markers with predictive power for DKD and MN were screened by a combination of a random forest model and fivefold cross-validation. POD-based ROC curves were completed in the training set (96 DKD and 98 MN samples) and testing set (33 DKD and 44 MN samples) to evaluate the diagnostic potential of the markers. A statistical analysis of the participants’ clinical data is shown in Table S1. Comparing the baseline characteristics of the two groups, we found that the eGFR in the DKD group was higher than that in the MN group (*p* < 0.001). Meanwhile, age, Cr, Alb, T/Cr, and T-CHO were lower in the DKD group (*p* < 0.05).

### Increased microbial diversity in the gut of DKD patients

**Figure 2.**
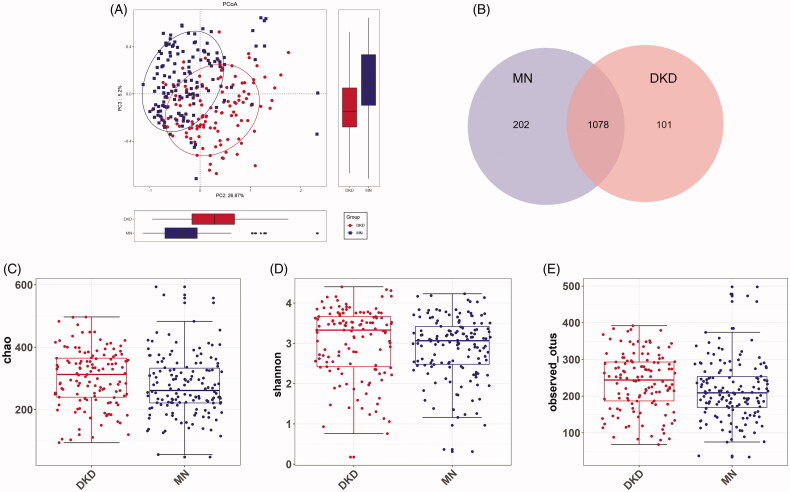
Microbial diversity. (A) PCoA analysis of the unweighted UniFrac distances revealed clustering of patients with DKD or MN (Adonis analysis: R2 = 0.06052, *p* = 0.001). (B) Venn diagram showing a considerable overlap of observed OTUs in both groups. The α diversity between the MN (*n* = 202) and DKD (*n* = 101) samples was estimated by (C) the Chao richness index, (D) the Shannon diversity index, and (E) the average number of observed OTUs. PCoA, principal coordinate analysis.

The rarefaction curve indicated that the sequencing depth of each sample approached the expected level, with an average of 30399 reads (maximum: 61603; minimum: 11285) (Figure S1A and Table S2). As shown by the rank-abundance/species-accumulation curves, estimated OTU richness and evenness were lower in DKD than in MN (Figure S1B, C). To visualize the structural diversity of the gut microbiome in the discovery group, we used PCoA/NMDS plots based on the unweighted UniFrac distances. The corresponding statistical significance of the beta diversity was measured separately by Adonis and ANOSIM. The results showed a significant difference in the gut microbiome in both groups (Adonis for PCoA: R^2^ = 0.06052, ANOSIM for NMDS: *R* = 0.2205, *p* < 0.001; Figure 2(A) and Figure S2A–C). Fewer total OTUs in the DKD group than in the MN group were observed in the Venn diagram (Figure 2(B)): 202 of 1280 OTUs were unique to MN, and 101 of 1179 OTUs were unique to DKD. The bacterial diversity in both groups was further estimated by the Ace/Chao-based richness index, Shannon diversity index and OTU index (*P* values = 0.0101/0.0077, 0.0492 and 0.0047, respectively; Figure 2(C–D) and Figure S2D). That is, the bacterial richness and diversity of DKD were significantly higher than those of MN.

### Altered microbial composition in DKD and MN

**Figure 3.**
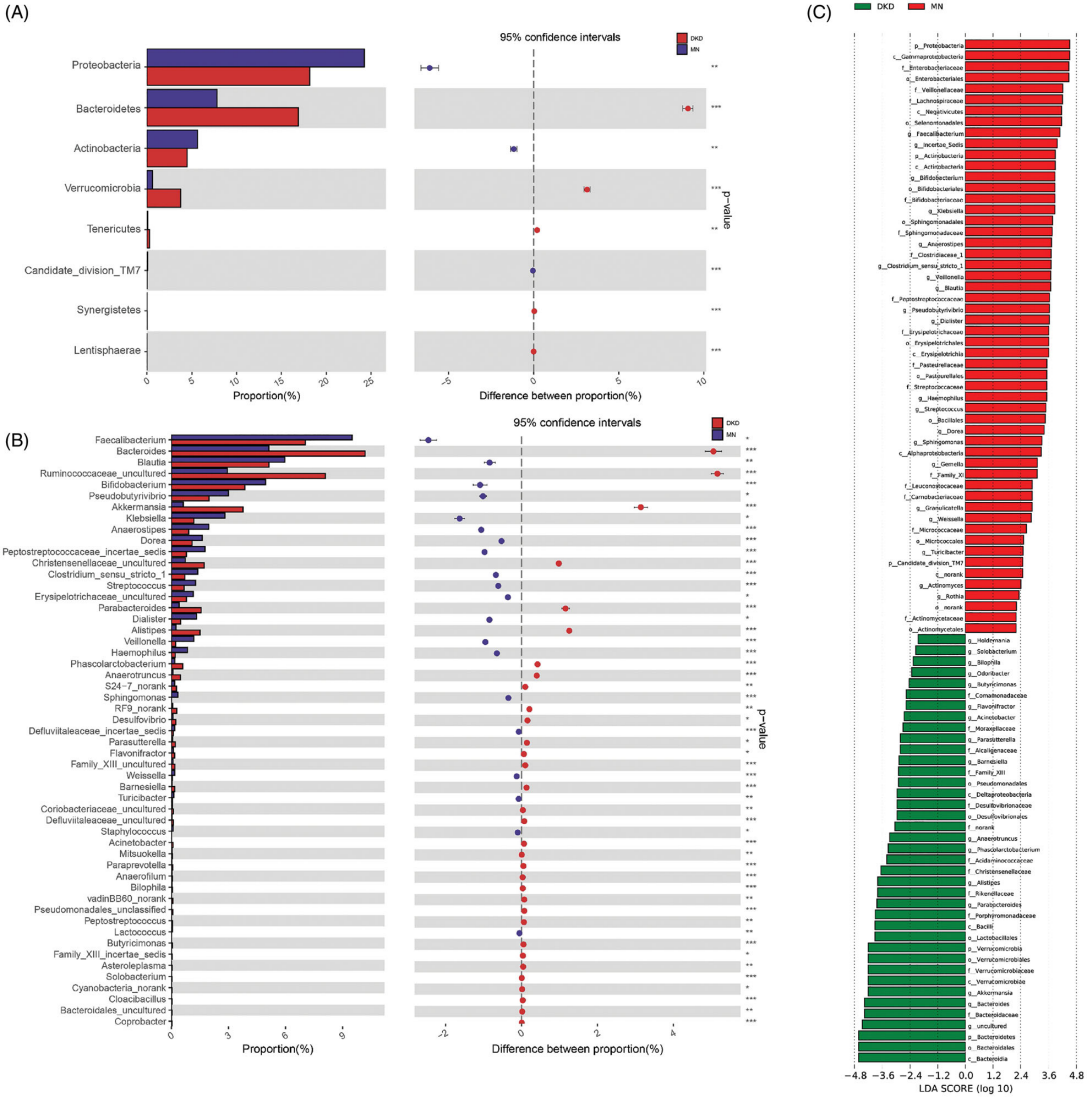
Variations in microbial profiles in the gut of DKD or MN patients. Differentially abundant microbial taxa at the phylum level (A) or genus level (B) by disease status are shown. At the phylum level, a *Bacteroidetes*-dominated microbial distribution was more common in DKD, and a *Proteobacteria*-dominated microbial distribution was more common in MN. The relative abundances of 33 among a total of 53 genera increased in DKD, and the remaining 20 were more abundant in MN. (C) LEfSe analysis based on the microbial taxonomic distributions of both groups (LDA > 2.0). LDA scores were logarithm-transformed. LEfSe, linear discriminant analysis (LDA) effect size.

The microbial profile of each sample at the phylum and genus levels is shown in Figure S3. As shown in Figure 3(A), the phylum-level microbial comparison identified eight differentially enriched gut microbiome phyla between the two groups, among which *Proteobacteria* was higher in MN (24.24% vs. 18.13%), *Bacteroidetes* was higher in DKD (16.88% vs. 7.82%), and the ratio of *Firmicutes/Bacteroidetes* was lower in DKD (3.31 vs. 7.84). The relative abundances of 53 genera differed according to the Wilcoxon rank-sum test. Of the genera enriched in DKD patients, *Peptostreptococcus* was classified as a potential pathogen, while 32 other genera represented probiotics. Conversely, the results in MN showed a higher abundance of pathobionts such as *Peptostreptococcaceae_incertae_sedis, Clostridium_sensu_stricto_1, Streptococcus, Veillonella, Haemophilus* and others, as well as several beneficial bacteria such as *Bifidobacterium, Lactococcus* and *Faecalibacterium* (Figure 3(B), Table S3). Microbial variation at the class, order, and family levels is shown in Figure S4.

To further validate these results, a LEfSe analysis of the bacterial community was performed on the sequencing data obtained from the discovery cohort. We confirmed that 93 gut microbiome taxa showed differential abundances; 39 of these taxa were abundant in DKD, and 53 were abundant in MN. Again, enrichment of various pathogens was observed in MN.

### OTU profiling of DKD and MN samples

**Figure 4.**
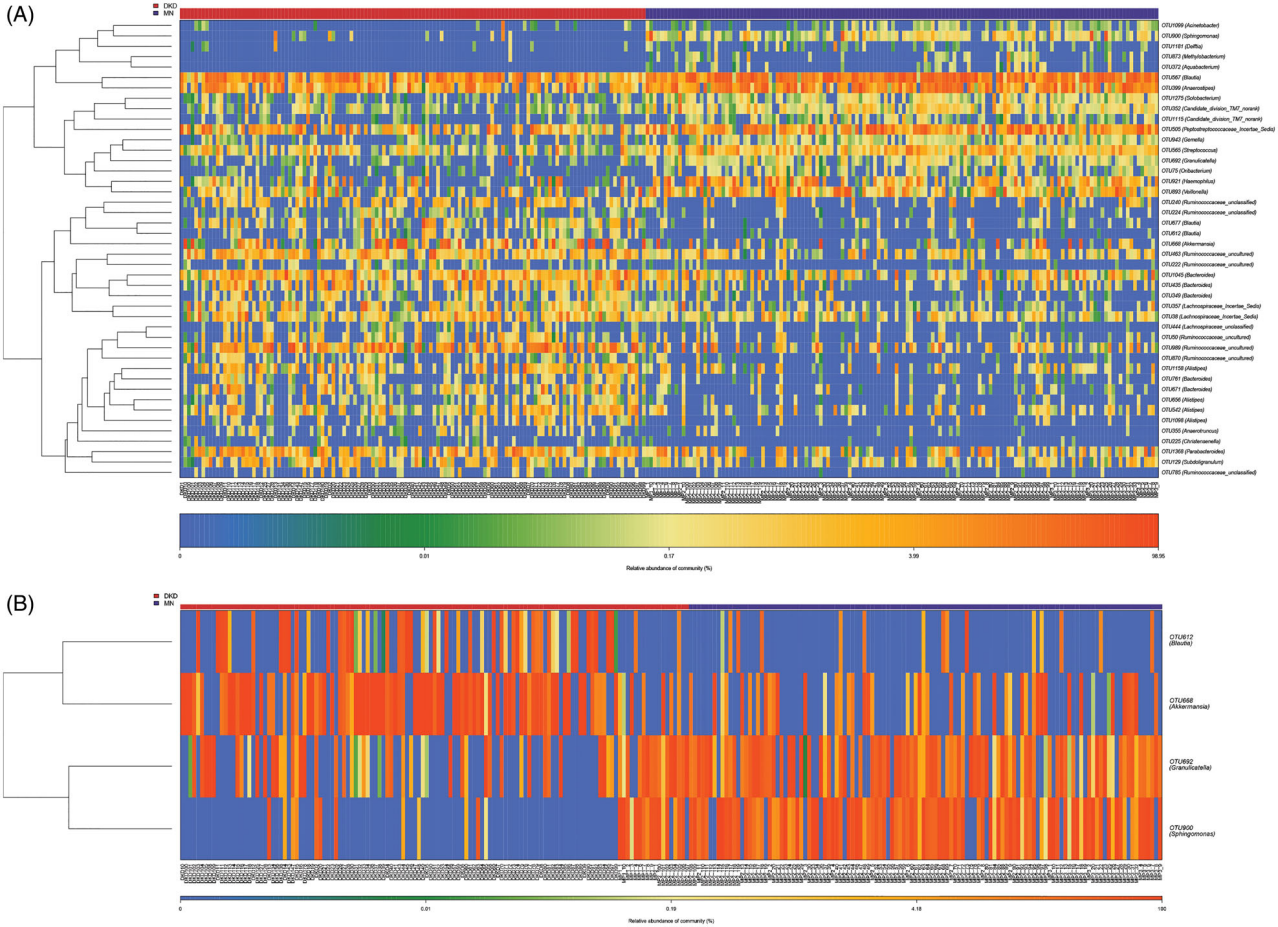
Identification of important microbial signatures for diagnostic model establishment. As shown in panel (A), the distributions of 45 key OTUs selected by the random forest model were strikingly different in the two disease groups. (B) Of these, four microbial OTUs were designated as putative markers to separate DKD from MN through fivefold cross-validation. OTUs belonging to *g_Sphingomonas* and *g_Granulicatella* were more enriched in MN, while OTUs belonging to *g_Blautia* and *g_Akkermansia* were more enriched in the DKD group. Notably, the distributions of these 4 OTUs in 22 samples (18 DKD and 4 MN) were excluded because they were undetectable.

We further performed OTU profiling of the DKD and MN samples to investigate potential microbiome-based markers that could best distinguish DKD from MN. The relative abundances of the top 50 OTUs in each sample are presented in Figure S5 and showed no significant difference between DKD and MN. Next, significantly different OTUs (with a *P* value < 0.05) were incorporated into the random forest model, which was used to identify key OTUs with the Wilcoxon rank-sum test. As a result, 44 key OTUs were selected and showed a symmetric distribution between DKD and MN (Figure 4 (A)). Finally, after five trials of fivefold cross-validation were performed on the selected OTUs, the combination of microbiome-based markers with the fewest OTUs (*n* = 4) and the minimum CV error rate were identified. The distribution of these 4 OTUs in each sample is shown in Figure 4(B). We noticed that two OTUs belonging to opportunistic pathogens, *g_Sphingomonas* and *g_Granulicatella*, were enriched in the MN group, while two other OTUs classified as beneficial bacteria, *g_Blautia* and *g_Akkermansia*, were relatively abundant in DKD samples.

### Evaluation of the diagnostic accuracy of the microbial markers

**Figure 5.**
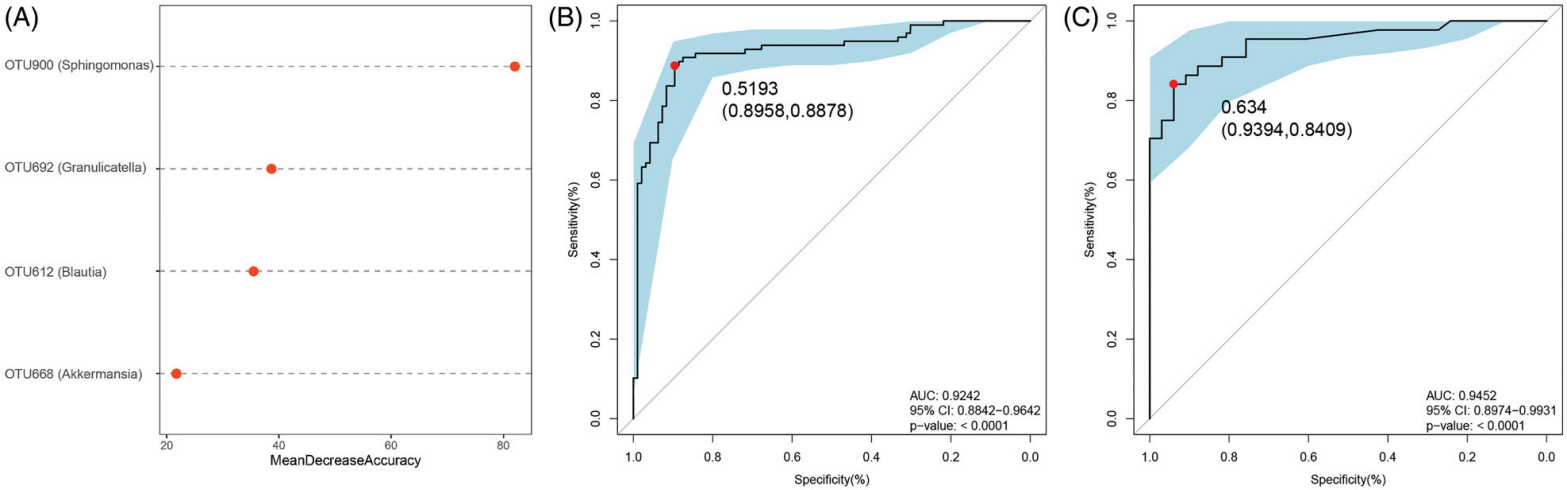
Differential predictive power of the microbial markers. (A) The relative importance of the four predictive markers was negatively correlated with the value of the mean decrease in accuracy. The ROC curves based on the microbial markers show the discrimination rates for DKD and MN in both the training (B) and testing sets (C). The AUC, 95% CI and *P* value are listed in the graph. ROC, receiver operating characteristic; AUC, area under the curve; CI, confidence interval.

To assess the diagnostic potential of the microbial biomarkers selected above, an ROC curve was constructed to distinguish DKD from MN. As mentioned above, the relative importance of the four microbiome-based markers was assigned by a random forest model (Figure 5(A)). We found that OTU900 could significantly improve predictive performance and had the highest importance (stability index: mean decrease of Gini >40.0, Figure S6). Using these 4 OTUs as biomarkers to separate 96 DKD samples from 98 MN samples, the AUC was 92.42% (95% confidence interval: 88.42%–96.42%; Figure 5(B)). Correspondingly, the POD index for MN was significantly higher than that for DKD in the training set (Figure S6B). Consistent with these results, 33 DKD and 44 MN samples were randomly partitioned into a testing set to validate the diagnostic potential of the markers for DKD and MN. The average POD value in patients with MN was significantly higher than that in patients with DKD (Figure S6C). The AUC in the testing set was 94.52%, and the 95% confidence interval (CI) was 89.74%−99.31% (Figure 5(C)). All these results indicated that microbiome-associated markers could be used as an alternative tool for distinguishing DKD from MN with high accuracy.

### Altered microbial functions in DKD and MN

**Figure 6.**
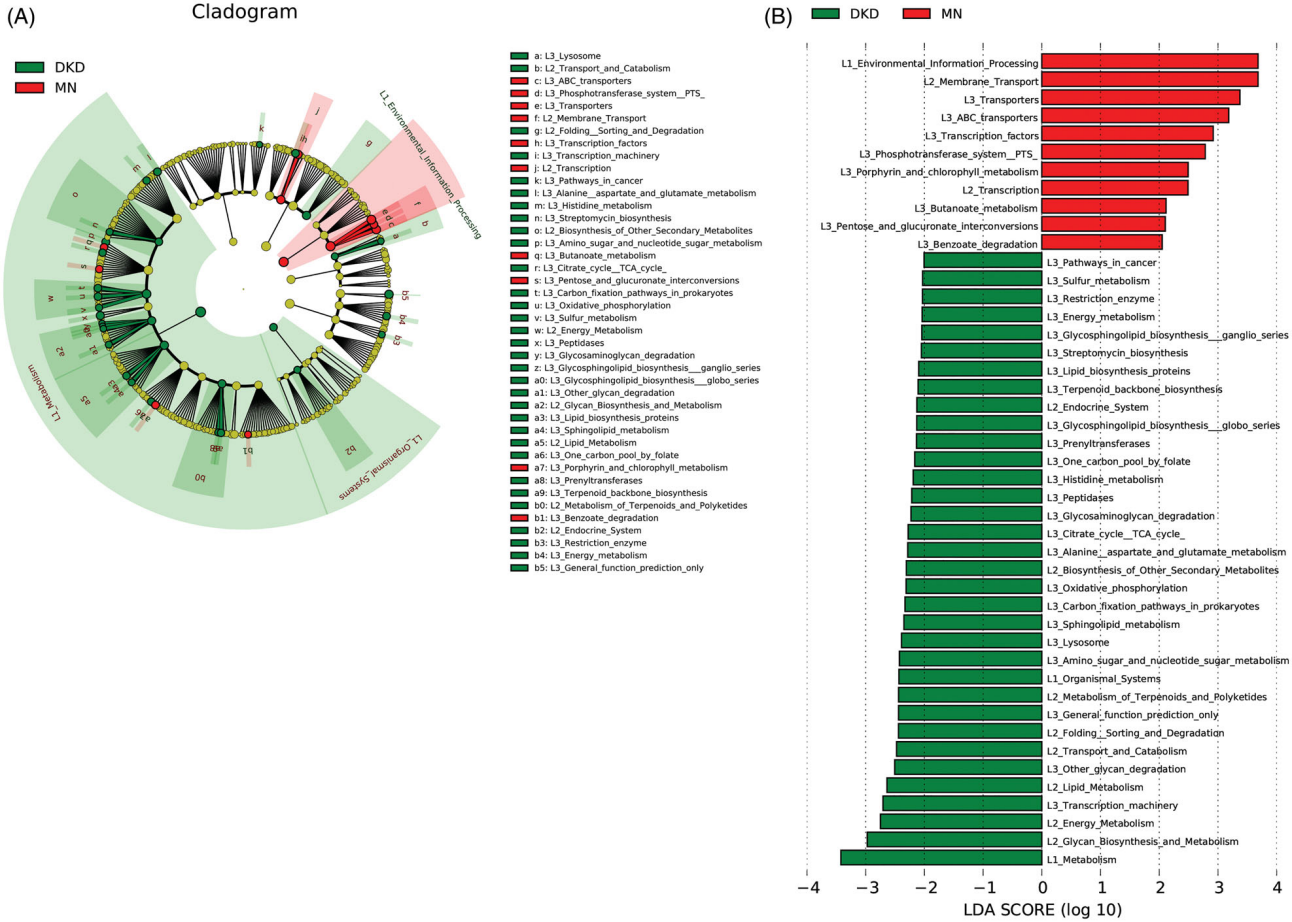
Prediction of the metabolic potential of the gut microbiome in DKD or MN. (A) PICRUSt revealed the taxonomic distribution of metabolic functions between DKD and MN. (B) A histogram of LDA scores showed 45 functional changes between DKD and MN. Of these, 34 were overexpressed in DKD, and 11 were overexpressed in MN. LDA, linear discriminant analysis.

We used LEfSe analysis to identify significant functional differences between DKD and MN using KEGG categories. PICRUSt identified 45 differentially abundant metabolic pathways between all 129 DKD and 142 MN samples (all LDA scores > 2.0, *p* < 0.05). Of these, we found overexpression of membrane transport involving ABC transporters and the phosphotransferase system (PTS) in the microbiome of MN compared to DKD; these pathways are related to the transportation of sugar and vitamin B12. Pentose and glucoronate interconversion were also highly enriched in MN. Furthermore, functional modules related to the metabolism of amino acids, such as alanine, aspartate, glutamate and histidine, were increased in the DKD microbiome. Additionally, greater enrichment of carbohydrate metabolism (amino sugar and nucleotide sugar metabolism), energy metabolism (oxidative phosphorylation) and lipid metabolism pathways were observed in DKD versus MN (Figure 6(A,B), Table S4).

## Discussion

Here, we applied the 16S rRNA sequencing technique to a large cohort consisting of 129 DKD and 142 MN samples to explore host-microbial interactions. Microbial analysis revealed that dysbiosis of the gut microbiome played a key role in the pathogenesis of DKD or MN. These significant differences in gut microbes could provide solid evidence for the classification of DKD and MN, exemplifying the concept of ‘microbial markers’ [22,33]. This study is the first to link the gut microbiome with DKD and MN, and here, we first attempt to use a noninvasive tool based on intestinal flora to distinguish DKD from MN.

Increasing evidence has suggested that gut microbiota composition and metabolism could be used as a diagnostic tool for diabetes [13], cardiovascular diseases [34] and other diseases [35]. For instance, Karlsson FH and his teammates developed a mathematical model based on microbial shotgun sequencing data to diagnose type-2 diabetes mellitus (T2DM) in European women and suggested a regional limitation of this model in the Chinese population [36]. Although a causal role of the gut microbiome in relation to microbial composition or metabolism in patients with ESRD or CKD has been reported many times [37], predictive tools based on genomic profiles have not been provided. In a previous study, DKD was reported as the leading cause of death in patients with ESRD, followed by MN, with a 40% contribution to ESRD-related mortality [4,5]. Moreover, the limitation of proteinuria in the early diagnosis of DKD [6,7] and the lack of options for PLA2R-negative MN identification need to be addressed further [11]. Undoubtedly, the analysis of the intestinal-microbiota in this study provides new insight into the identification and classification of DKD and MN. Here, we provided solid evidence that microbial markers based on 4 OTUs could separate MN samples from DKD samples with a high accuracy of 92.42%, strongly supported by the 94.52% AUC in the interval validation cohort.

We found that bacterial alpha diversity was increased in patients with MN compared to those with DKD. Either the relative increase in beneficial bacteria producing SCFAs in the DKD group or the relative depletion of such probiotics in MN could explain this alteration. For instance, patients with DKD exhibited significantly higher proportions of the genera *Bifidobacterium, Parabacteroides* and *Butyricimonas* compared with MN patients (Table S3). Recent studies have reported that the gut microbiome could promote disease development, which was characterized by reduced diversity [17,38–40]. In a metagenomewide association study of patients with diabetes, microbial dysbiosis could be summarized as a depletion of some butyrate-producing bacteria [12]. Analogous to the results in diabetes, the greater bacterial richness or diversity observed in DKD was likely to suggest a greater reduction of beneficial bacteria in MN rather than an increase in DKD. Additionally, SCFAs provide an energy source for intestinal epithelial cells [41], a decrease in which could promote intestinal mucosal injury, thereby providing a mechanism for the involvement of bacteria in the initiation of DKD/MN [42–44]. The proportions of opportunistic pathogens such as *Peptostreptococcaceae_incertae_sedis*, *Clostridium_sensu_stricto_1, Streptococcus, Veillonella, Haemophilus* and others were increased in MN. These bacteria, accompanied by their production of lipopolysaccharides (LPS), could be translocated to the bloodstream through the impaired gut, further activating the NF-κB pathway, triggering the release of proinflammatory factors (e.g. TNF, IL-1, IL-6), and thereby promoting the development of inflammatory diseases [27,42,45]. In summary, a depletion of SCFA-producing genera and an increase in pathobionts suggested more severe immune impairment in patients with MN. Notably, compensatory increases in mucin-degrading bacteria such as *Akkermansia* [46–48] and beneficial bacteria (*Lactococcus, Faecalibacterium* and *Pseudobutyrivibrio*) in DKD were also observed in our study.

There was no significant difference in the abundance of the phylum *Firmicutes* between the groups, while the relative abundances of *Proteobacteria* and *Bacteroidetes* were increased in MN and DKD, respectively. *Firmicutes* plays a key role in SCFA production and could be considered a therapeutic target to inhibit the development of T2DM [18,49]. *Proteobacteria* and *Bacteroidetes*, major bacterial phyla that produce LPS, were enriched, indicating an unhealthy host status. The ratio of *Firmicutes/Bacteroidetes* (F/B) was also calculated and compared between the two groups (DKD vs. MN: 3.31 vs. 7.84). Research has found that a typical F/B ratio for adults is 10.9 [50]. In our study, the F/B ratios in both DKD and MN were abnormal, indicating inferior host health.

The potential functions of the gut microbiome in DKD/MN were predicted using the KEGG database. We found an increase in the aerobic metabolism module, containing amino acid metabolism (alanine, aspartate and glutamate) and the citrate cycle; this result indicated the potential of the microbial environment to shift toward more aerotolerant conditions (Table S4). We also observed overexpression of histidine metabolism in DKD; its product histamine might be involved in mediating the inflammatory response [51]. ABC transporters mediate the transport of hydrophilic compounds and promote the growth of various bacteria, such as *Mycobacterium tuberculosis* [52]. The PTS is involved in the phosphorylation of sugars [53], and the interconversion of pentose and glucoronate provides substrates for the synthesis of nucleic acids and maintains the reducing state of glutathione. Higher enrichment of these three KEGG pathways was observed in MN patients, and their roles in MN development remain to be determined.

Overall, we observed significant differences in microbial composition and function in the gut of DKD or MN patients. Particularly, microbial profiles based on only four OTUs could be used to construct a diagnostic model with high accuracy, which might be used as a noninvasive option to distinguish DKD from MN. This project was a new attempt to identify different diseases on the basis of the gut microbiome. It also provided the possibility to distinguish different nephropathies using gut microbial biomarkers. Further studies should evaluate the effects of fecal metabolites on different disease statuses and assess the host response to microbiota-targeted therapies, with the hope of delaying disease progression.

## Supplementary Material

Supplemental Material

Supplemental Material

Supplemental Material

Supplemental Material

Supplemental Material

Supplemental Material

Supplemental Material

Supplemental Material

Supplemental Material

Supplemental Material
