# Supplementary material for: The gut microbiome in differential diagnosis of diabetic kidney disease and membranous nephropathy
Source: Ren Fail. 2020 Oct 30;42(1):1100–10. doi: 10.1080/0886022X.2020.1837869 (PMC7599019; doi:10.1080/0886022X.2020.1837869)
Supplement: Supplemental Material [file IRNF_A_1837869_SM5519.doc]

| Clinical variables | All individuals | | P |
| --- | --- | --- | --- |
|  | DKD (n=129) | MN(n=142) |  |
| Age[Year] | 56(49，65) | 49(43，56) | <0.001 |
| Gender |  | | 0.939 |
| Female(%) | 46(35.7%) | 50(35.2%) |  |
| Male(%) | 83(64.3%) | 92(64.8%) |  |
| eGFR (mL /min) | 56.42(25.49,96.62) | 100.53(84.17,108.29) | <0.001 |
| Cr (μmol/L) | 107.0(71.0,214.2) | 72.0(61.0,86.25) | <0.001 |
| Alb (g/L) | 34.83+/-8.11 | 31.43+/-7.60 | <0.001 |
| 24h-pro(g) | 3.02(0.55,6.38) | 1.83(0.53,4.87) | 0.025 |
| T/Cr (g/g) | 3.92(1.51,8.67) | 0.79(0.19,2.72) | <0.001 |
| T-CHO(mmol/L) | 5.18(3.99,6.59) | 4.38(3.26,5.49) | <0.001 |
| TG(mmol/L) | 1.88(1.24,2.78) | 1.47(1.10,2.14) | 0.002 |

Table S1. Baseline characteristics of participants

K-S test was used to characterize the normality of two groups. One-way ANOVA analysis was used to assess whether the variance was equal between the two groups. Exploiting t test to compare continuous variables, while Wilcoxon rank-sum test for discontinuous variables and Chi-square test for categorical variables. Statistical analyses were performed using SPSS V .26.0.
