# Supplementary figures and images for: The gut microbiome in differential diagnosis of diabetic kidney disease and membranous nephropathy
Source: Ren Fail. 2020 Oct 30;42(1):1100–10. doi: 10.1080/0886022X.2020.1837869 (PMC7599019; doi:10.1080/0886022X.2020.1837869)

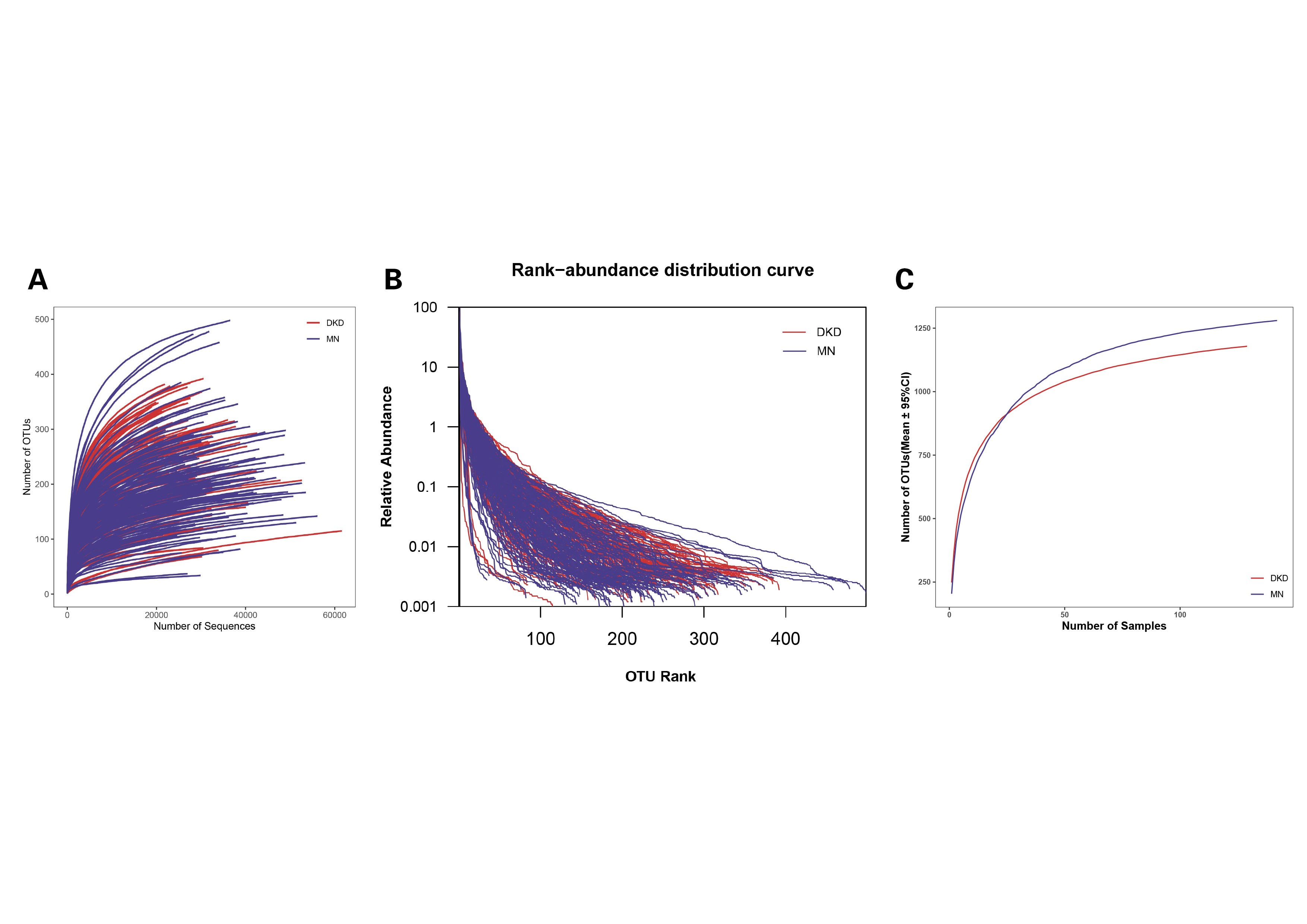

Supplement: Supplemental Material [file IRNF_A_1837869_SM5466.tif]

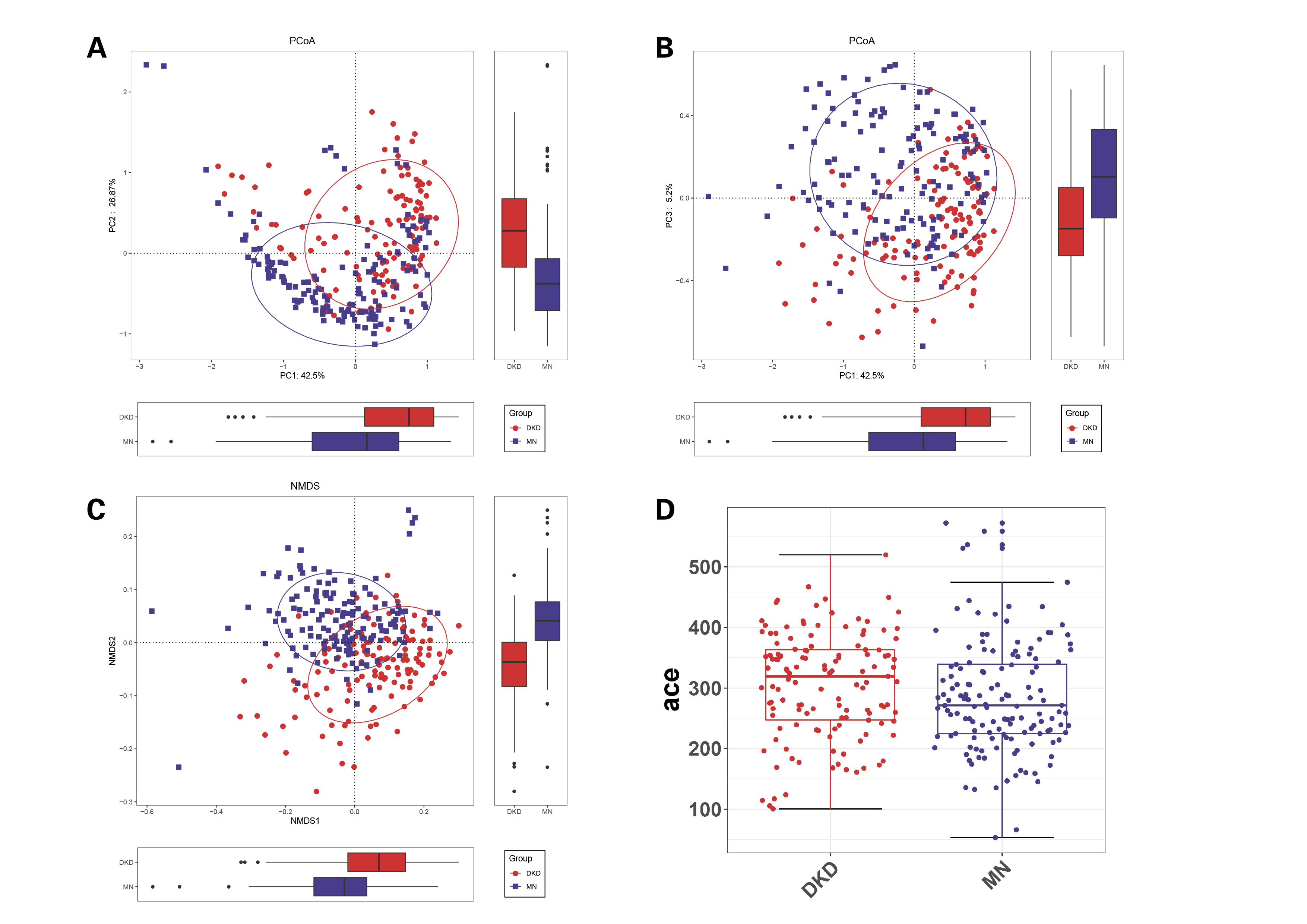

Supplement: Supplemental Material [file IRNF_A_1837869_SM5452.tif]

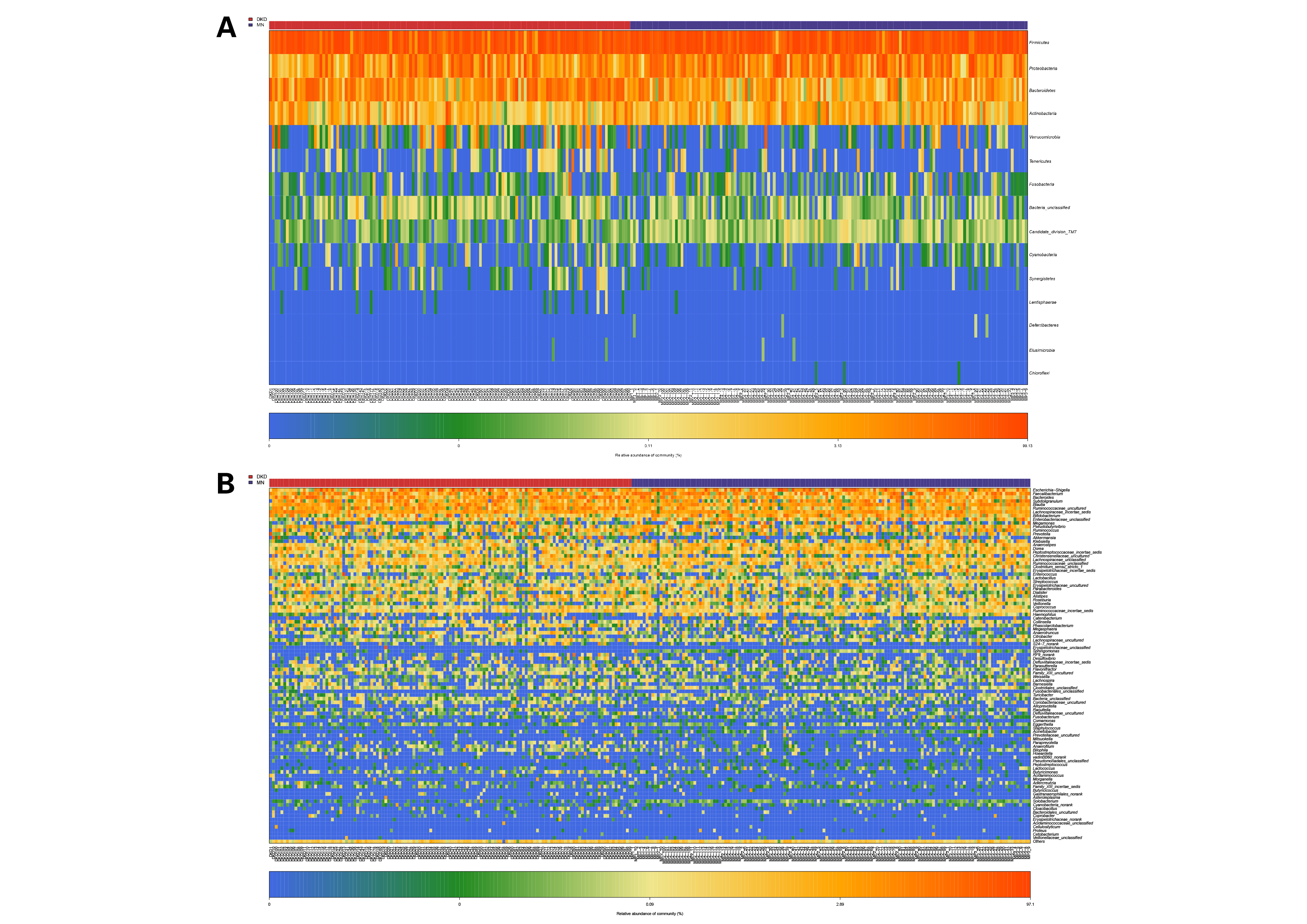

Supplement: Supplemental Material [file IRNF_A_1837869_SM5431.tif]

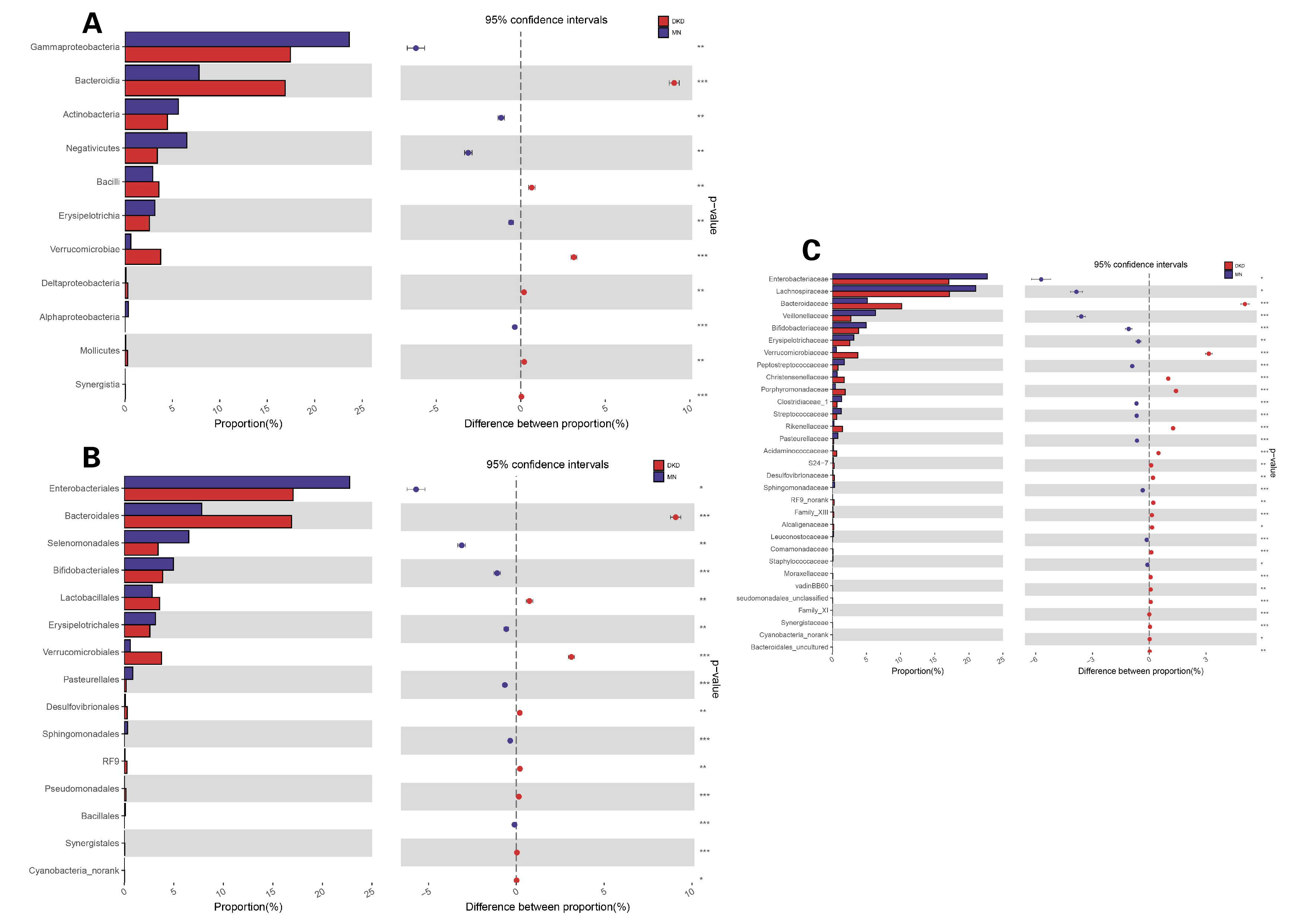

Supplement: Supplemental Material [file IRNF_A_1837869_SM5409.tif]

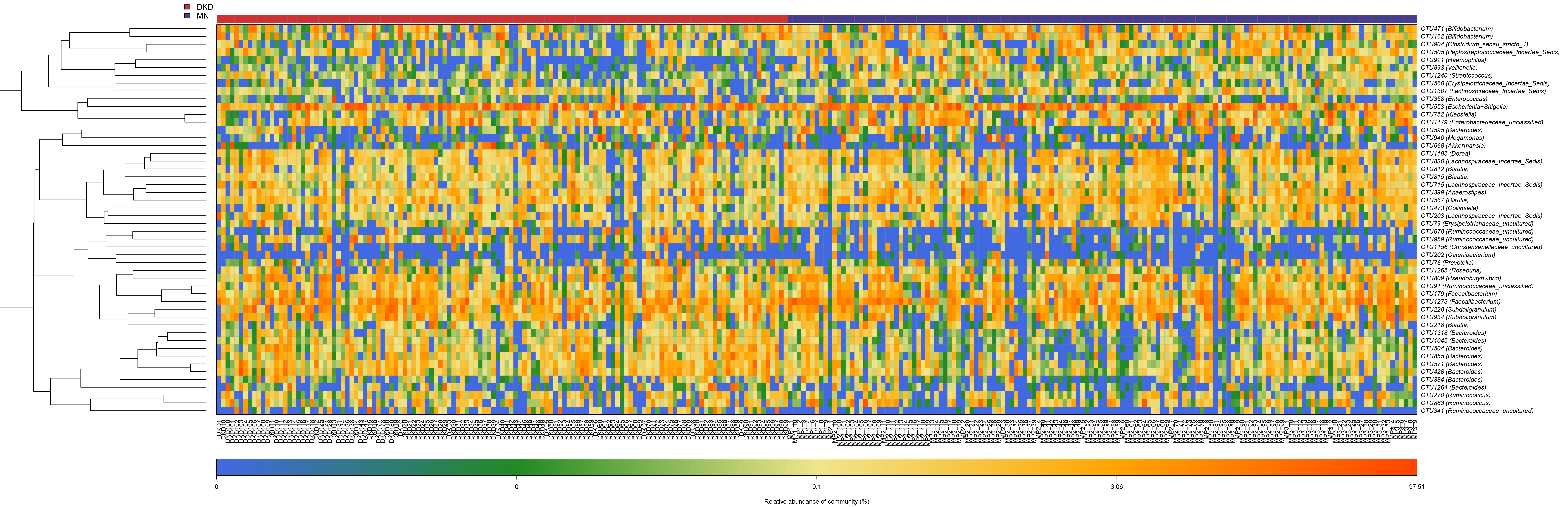

Supplement: Supplemental Material [file IRNF_A_1837869_SM5353.tif]

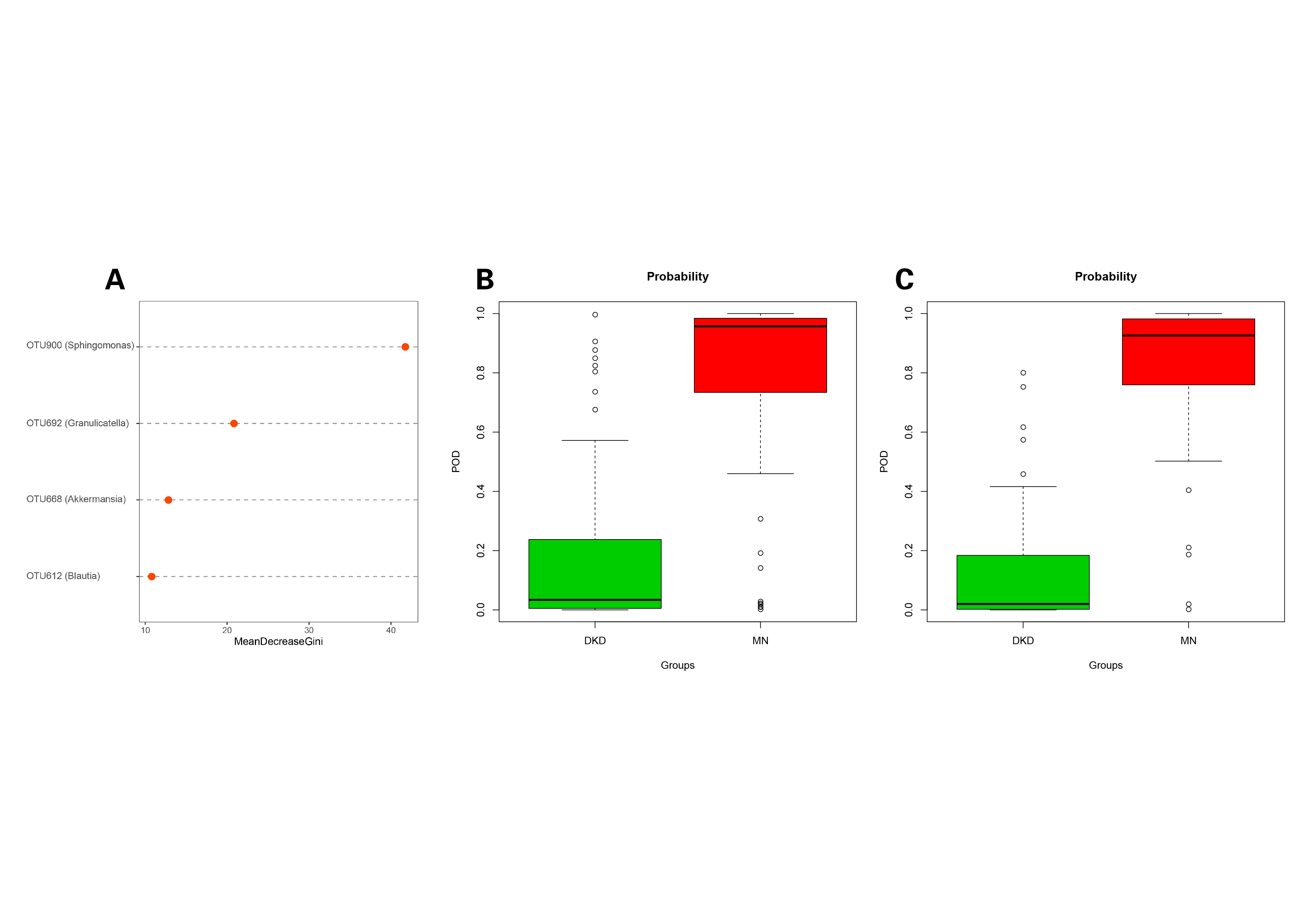

Supplement: Supplemental Material [file IRNF_A_1837869_SM5296.tif]
